# Trends in renal calculus composition and 24-hour urine analyses in patients with neurologically derived musculoskeletal deficiencies

**DOI:** 10.1590/S1677-5538.IBJU.2018.0531

**Published:** 2019-07-27

**Authors:** Lee A. Hugar, Ilan Kafka, Thomas W. Fuller, Hassan Taan, Timothy D. Averch, Michelle J. Semins

**Affiliations:** 1 Department of Urology, University of Pittsburgh School of Medicine, Pittsburgh, PA, USA; 2 Shaare Zedek Medical Center, Jerusalem, Israel

**Keywords:** Nephrolithiasis, Urinary Bladder, Neurogenic, Kidney Calculi

## Abstract

**Purpose:**

To better characterize metabolic stone risk in patients with neurologically derived musculoskeletal deficiencies (NDMD) by determining how patient characteristics relate to renal calculus composition and 24-hour urine parameters.

**Materials and Methods:**

We performed a retrospective cohort study of adult patients with neurologically derived musculoskeletal deficiencies presenting to our multidisciplinary Kidney Stone Clinic. Patients with a diagnosis of NDMD, at least one 24-hour urine collection, and one chemical stone analysis were included in the analysis. Calculi were classified as primarily metabolic or elevated pH. We assessed in clinical factors, demographics, and urine metabolites for differences between patients who formed primarily metabolic or elevated pH stones.

**Results:**

Over a 16-year period, 100 patients with NDMD and nephrolithiasis were identified and 41 met inclusion criteria. Thirty percent (12 / 41) of patients had purely metabolic calculi. Patients with metabolic calculi were significantly more likely to be obese (median body mass index 30.3kg / m^2^ versus 25.9kg / m^2^), void spontaneously (75% vs. 6.9%), and have low urine volumes (100% vs. 69%). Patients who formed elevated pH stones were more likely to have positive preoperative urine cultures with urease splitting organisms (58.6% vs. 16.7%) and be hyperoxaluric and hypocitraturic on 24-hour urine analysis (37mg / day and 265mg / day versus 29mg / day and 523mg / day).

**Conclusions:**

Among patients with NDMD, metabolic factors may play a more significant role in renal calculus formation than previously believed. There is still a high incidence of carbonate apatite calculi, which could be attributed to bacteriuria. However, obesity, low urine volumes, hypocitraturia, and hyperoxaluria suggest an underrecognized metabolic contribution to stone formation in this population.

## INTRODUCTION

Neurologically derived musculoskeletal deficiencies (NDMD) encompass a heterogeneous group of etiologies for neurogenic bladder such as spinal cord injury (SCI), spina bifida (SB), cerebrovascular accident (CVA), and cerebral palsy (CP). The incidence of renal calculi in NDMD patients mirrors that of the general population. Among NDMD patients, however, renal calculi present in a delayed or atypical manner and account for a disproportionate number of urologic procedures (1, 2). Compounding the morbidity associated with renal calculus disease is an increased risk of urinary tract colonization and infection due to neurogenic bladder. Because of this, patients with NDMD face a high risk of developing elevated pH or “infection-related” calculi.

A mainstay of care for patients with high risk and recurrent renal calculus disease is the 24-hour urine collection, but its use for patients with NDMD is controversial. In fact, 24-hour urine collection has been recommended against in subsets of the NDMD population given a low occurrence of metabolic abnormalities and the belief that these calculi are of “pure infectious” etiology (3, 4). These beliefs have been challenged by a growing body of literature detailing a shift in renal calculus composition, including staghorn calculi, toward metabolically derived calculi in the NDMD population (5–7). Regardless, there are no clear guidelines for the use of 24-hour urine collection and medical management of renal calculi in NDMD patients (8).

For these reasons, we performed the following study to better characterize metabolic stone risk in the NDMD population by determining how patient characteristics relate to renal calculus composition and 24-hour urine parameters. We were also interested in the association between renal calculus composition and type of neurologic deficiency and bladder management strategy. Our overall goal is to work towards determining best practices for the use of 24-hour urine collection and directed medical management in patients with NDMD and renal calculus disease.

## MATERIALS AND METHODS

### Study design and cohort overview

We performed a retrospective cohort study from January 2000 through December 2016. We screened adult patients 18 years and older for the presence of NDMD who presented consecutively to our multidisciplinary Kidney Stone Clinic. This specialty clinic is held monthly, specifically for patients with complex renal calculus disease. Disciplines present for Kidney Stone clinic include urology, nephrology, endocrinology, and dietetics. Patients are initially seen by a urologist, who guides surgical or medical management, and are subsequently evaluated by one of our medical colleagues as needed.

Neurological lesions considered to be NDMD included SCI, SB, CVA, neurodegenerative diseases (e.g. multiple sclerosis and Parkinson's disease), and CP. Inclusion criteria consisted of diagnosis of an NDMD, at least one 24-hour urine collection, and one chemical calculus analysis. Patients were excluded if they lacked a neurologic diagnosis, 24-hour urine study, or chemical calculus analysis. Patients not followed in the multidisciplinary stone clinic were also excluded from analysis. The study was approved by the Institutional Review Board at the University of Pittsburgh (PRO15060101).

### Data acquisition

Collected variables included age, sex, body mass index (BMI), bladder management strategy, relevant comorbidities (e.g. diabetes mellitus, chronic kidney disease, thyroid and parathyroid disorders, and structural and anatomic genitourinary abnormalities), type of neurological lesion, preoperative urine culture, and presence and type of directed medical management. We also recorded renal calculus composition and 24-hour urine collection parameters via Litholink (Chicago, IL, USA). All chemical stone analyses were performed via infrared spectroscopy and outsourced to a private lab (Quest Diagnostics, Madison, New Jersey, USA). For patients with multiple 24-hour urine studies, we used the study submitted most closely to that patient's first renal calculus chemical analysis.

### Definition of variables

Primary calculus classification was determined by the crystal comprising 50% or more of the calculus on chemical analysis. Calculi were classified as primarily metabolic or elevated pH and further classified as pure or mixed (i.e. consisting of both metabolic and elevated pH components). Calculi classified as elevated pH contained either carbonate apatite or magnesium ammonium phosphate crystals. In order to obtain the best baseline urine metabolic profile, we recorded urine parameters from the 24-hour urine collection most temporally proximate to the patient's first chemical stone analysis. All parameters were analyzed as both continuous and categorical variables, with cut points based on established definitions of urine metabolites (9).

### Statistical analysis

Categorical variables were analyzed using the Chi squared test or Fisher's exact test when appropriate. Continuous variables were analyzed using the Student's t-test. The threshold for statistical significance was set at a two-tailed p--value < 0.05.

## RESULTS

**Figure 1:**
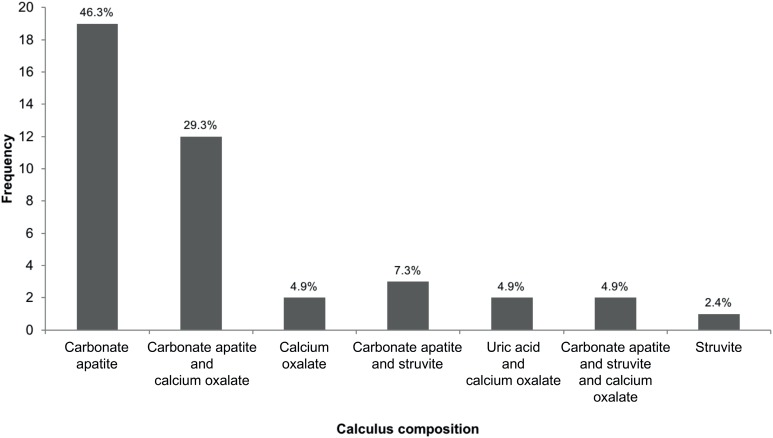
Frequency of calculus compositions.

Over a 16-year period, 100 patients with NDMD and nephrolithiasis were identified. Of these, 41 had both a chemical calculus analysis and 24-hour urine collection. The proportion of each unique calculus composition is shown in Figure-1. The majority of calculi were classified as elevated pH (70%, 29 / 41), but one third of the cohort had primarily metabolic calculi. Most elevated pH calculi were pure stones (69%, 20 / 29), while metabolic calculi were mostly mixed (75%, 9 / 12).

Demographic and clinical characteristics stratified by primary calculus classification are shown in Table-1. Age, sex, and neurological lesion were evenly distributed between elevated pH and metabolic stone formers. Patients with metabolic calculi were significantly more likely to be obese, with a median BMI of 30.3 compared to 25.9 (p = 0.01). Bladder management also differed between groups, with elevated pH stone former dependent on clean intermittent catheterization or an indwelling catheter. Metabolic stone formers were more likely to void spontaneously (p = 0.001). Over 75% of the cohort had positive preoperative urine cultures and nearly 50% of cultures had urease splitting organisms, with both being more common in elevated pH stone formers (86% vs. 50% and 59% vs. 17%, respectively).

**Table 1:** Demographic and clinical characteristics overall and by primary calculus classification.

Characteristics		Overall n = 41	Metabolic n = 12	Elevated pH n = 29	p-value
Age, years, median (IQR)		49.0 (27.0)	43.5 (21.5)	50.0 (33.0)	**0.90**
**Sex**					**0.8**
	Male, n (%)	22 (54)	6 (50)	16 (55.2)	
	Female, n (%)	19 (46)	6 (50)	13 (44.8)	
Body mass index, kg/m^2^, median (IQR)		28.6 (9.0)	30.3 (7.0)	25.9 (9.8)	**0.04**
**Bladder management**					**0.001**
	Spontaneously void, n (%)	11 (26.8)	9 (75.0)	2 (6.9)	
	Clean intermittent catheterization, n (%)	11 (26.8)	2 (16.7)	9 (31.0)	
	Indwelling catheter, n (%)	16 (39.0)	1 (8.3)	15 (51.7)	
	Ileal conduit, n (%)	3 (7.3)	0 (0.0)	3 (10.3)	
Positive preoperative urine culture, n (%)		31 (75.6)	6 (50.0)	25 (86.2)	**0.04**
Culture with urea-splitting organism, n (%)		19 (46.3)	2 (16.7)	17 (58.6)	**0.01**
**Neurological lesion**					**0.1**
	Paraplegia	5 (12.2)	2 (16.7)	3 (10.3)	
	Quadriplegia	9 (22.0)	1 (8.3)	8 (27.6)	
	Spina bifida	7 (17.1)	1 (8.3)	6 (20.7)	
	Cerebrovascular	5 (12.2)	4 (33.3)	1 (3.5)	
	Cerebral palsy	5 (12.2)	2 (16.7)	3 (10.3)	
	Neurodegenerative	10 (24.4)	2 (16.7)	8 (27.6)	

Abbreviations: **IQR** = interquartile range; Percentages may not add to 100 due to rounding

Neither primary calculus classification nor sex were associated with the total number of metabolic abnormalities or the proportion of patients on directed medical therapy (all p > 0.05). However, elevated pH stone formers were less likely to have low urine volumes and more likely to be both hyperoxaluric and hypocitraturic (both p < 0.05, show in Tables 2 and 3). Male stone formers were more likely to be hypernatrituric (p < 0.03) and hyperoxaluric (p < 0.05). Neurological lesion was not associated with any urinary parameters (all p > 0.05).

**Table 2:** Urinary metabolic parameters by primary calculus classification.

Characteristic		Metabolic n = 12	Elevated pH n = 29	p-value
Metabolic abnormalities, median (IQR)		4.5 (2.5)	4 (2.0)	0.3
Current directed medical therapy, n (%)		5 (41.7)	5 (17.2)	0.1
**Volume (L), median (IQR)**		**1.5 (0.8)**	**1.6 (2)**	**0.04**
	Low volume, n (%)	12 (100.0)	20 (69.0)	0.04
**Supersaturation calcium oxalate, median (IQR)**		**6.4 (5.4)**	**4.5 (4.2)**	**0.2**
	High supersaturation, n (%)	3 (25.0)	3 (10.3)	0.3
**Urine calcium (mg/day), median (IQR)**		**163 (171.5**)	**116 (102.0)**	**0.3**
	Hypercalciuria, n (%)	3 (25.0)	2 (6.9)	0.1
	Hypercalciuria with hypernatrituria, n (%)	1 (8.3)	1 (3.5)	0.5
**Urine sodium (mmol/d), median (IQR)**		**139 (55.5)**	**104 (65.0)**	**0.4**
	Hypernatrituria, n (%)	5 (41.7)	7 (24.1)	0.3
**Urine oxalate (mg/day), median (IQR)**		**29 (11.5)**	**37 (27.0)**	**0.05**
	Hyperoxaluria, n (%)	1 (8.3)	7 (24.1)	0.4
**Urine citrate (mg/day), median (IQR)**		**523 (439.0)**	**265 (302.0)**	**0.9**
	Hypocitraturia, n (%)	3 (25.0)	17 (58.6)	0.05
	pH (continuous), median (IQR)	6.8 (1.4)	6.8 (1.3)	0.5
**pH (categorical)**				0.3
	pH <6, n (%)	3 (25.0)	3 (10.3)	
	pH 6-7, n (%)	3 (25.0)	14 (48.3)	
	pH >7, n (%)	6 (50.0)	12 (41.4)	
**Supersaturation calcium phosphate, median (IQR)**		**1.3 (2.7)**	**0.9 (1.0)**	**0.1**
	High supersaturation, n (%)	5 (41.7)	6 (21.4)	0.3
**Supersaturation uric acid, median (IQR)**		**0.2 (0.8)**	**0.1 (0.2)**	**0.4**
	High supersaturation, n (%)	3 (25.0)	3 (10.3)	0.3
**Urine uric acid (g/d), median (IQR)**		**0.5 (0.2)**	**0.4 (0.3)**	**0.7**
	Hyperuricosuria, n (%)	1 (8.3)	4 (14.3)	1.0
**Urine magnesium (mg/d), median (IQR)**		**75 (46.5)**	**71 (86.0**)	**0.2**
	Low magnesium, n (%)	2 (16.7)	7 (24.1)	0.7
**Urine creatinine (mg/kg/d), median (IQR)**		**13 (6.0)**	**12 (6.9)**	**0.9**
	Low urine creatinine, n (%)	8 (66.7)	21 (75.0)	0.7

Abbreviations: **IQR** = interquartile range

**Table 3:** Urinary metabolic parameters by sex.

Characteristic		Female n = 19	Male n = 22	p-value
Metabolic abnormalities, median (IQR)		4 (3.0)	4 (2.0)	0.3
Current directed medical therapy, n (%)		5 (26.3)	5 (22.7)	1.0
**Volume (L), median (IQR)**		**1.5 (0.8)**	**1.9 (1.9)**	**0.2**
	Low volume, n (%)	17 (89.5)	15 (68.2)	0.1
**Supersaturation calcium oxalate, median (IQR)**		**5.0 (3.8)**	**5.4 (4.4)**	**1.0**
	High supersaturation, n (%)	3 (15.8)	3 (13.6)	1.0
**Urine calcium (mg/day), median (IQR)**		**103 (65.0)**	**158 (150.0)**	**0.07**
	Hypercalciuria, n (%)	2 (10.5)	3 (13.6)	1.0
	Hypercalciuria with hypernatrituria, n (%)	0 (0.0)	2 (9.1)	0.5
**Urine sodium(mmol/d), median (IQR)**		**105(69)**	**132 (87)**	**0.03**
	Hypernatrituria, n (%)	2 (10.5)	10 (45.5)	0.02
**Urine oxalate (mg/day), median (IQR)**		**26 (20.0)**	**37 (26.0)**	**0.05**
	Hyperoxaluria, n (%)	1 (5.3)	7 (31.8)	0.05
**Urine citrate (mg/day), median (IQR)**		**275 (445.0)**	**395 (451.0)**	**0.8**
	Hypocitraturia, n (%)	11 (57.9)	9 (40.9)	0.3
	pH (continuous), median (IQR)	6.9 (1.3)	7.0 (1.1)	0.4
**pH (categorical)**				0.5
	pH <6, n (%)	4 (21.1)	2 (9.1)	
	pH 6-7, n (%)	8 (42.1)	9 (40.9)	
	pH >7, n (%)	7 (36.8)	11 (50.0)	
**Supersaturation calcium phosphate, median (IQR)**		**0.7 (1.9)**	**1.0 (1.5**)	**0.3**
	High supersaturation, n (%)	5 (26.3)	6 (28.6)	0.9
**Supersaturation uric acid, median (IQR)**		**0.1 (0.2)**	**0.1 (0.2)**	**0.3**
	High supersaturation, n (%)	4 (21.1)	2 (9.1)	0.4
**Urine uric acid (g/d), median (IQR)**		**0.4 (0.3)**	**0.5 (0.2)**	**0.1**
	Hyperuricosuria, n (%)	1 (5.3)	4 (19.1)	0.3
**Urine magnesium(mg/d), median (IQR)**		**51 (61)**	**90 (105.0)**	**0.3**
	Low magnesium, n (%)	3 (15.8)	6 (27.3)	0.5
**Urine creatinine (mg/kg/d), median (IQR**)		**12 (6.2)**	**14 (7.4)**	**0.1**
	Low urine creatinine, n (%)	13 (72.2)	16 (72.7)	1.0

Abbreviations: **IQR** = interquartile range

## DISCUSSION

In a cohort of patients with NDMD and nephrolithiasis, the majority formed elevated pH calculi. Half of the cohort formed calculi harboring a metabolic component (51%, 21 / 41) and nearly one third formed primarily metabolic calculi (29%, 12 / 41). This reinforces bacteriuria as an important risk factor for calculus formation and suggests that metabolic abnormalities may be an under-recognized modifiable risk factor for renal calculus disease in the NDMD population. In addition, significant differences existed between patients that form elevated pH versus metabolic calculi. These differences-such as BMI, bladder management, 24-hour urine volume, urinary oxalate, and urinary citrate-may guide medical management of nephrolithiasis patients with NDMD.

Renal calculus composition and its etiology in patients with neurological deficits are more complicated than previously thought. Recent studies have shown increasing rates of metabolic stones in portions of the NDMD population (6). Despite this, the metabolic work up and management of nephrolithiasis in patients at risk of elevated pH or “infection stones” remains controversial. Past studies have argued against the use of 24-hour urine collection analysis in patients with infected renal calculi (3, 4, 10, 11). However, we found significant metabolic differences in our cohort of patients with NDMD.

There are a number of potentially important differences between NDMD patients that form primarily elevated pH versus metabolic calculi. First, patients who form primarily metabolic calculi were more likely to be obese, suggesting that obesity may drive and exacerbate their risk of metabolic calculi formation. Indeed, obesity has been reported to be an independent risk factor for nephrolithiasis in otherwise healthy adults due lithogenic factors in the urine; such as urinary sodium, oxalate, and uric acid (12–16). However, metabolic stone formers in our study had significantly lower oxalate, reinforcing the complex nature of calculus disease in NDMD patients. Second, we found that patients forming primarily elevated pH calculi were more likely to have indwelling catheters or perform clean intermittent catheterization and have a higher rate of positive preoperative urine culture. Urinary tract colonization and bladder management have long been recognized as risk factors for calculus formation in patients with spinal cord injury (17, 18). Our study reinforces this in a more heterogeneous cohort of patients with neurological deficits.

Patients with NDMD that form different primary stone types also have significant differences in 24-hour urine study parameters. We found that patients with metabolic calculi were more likely to have lower 24-hour urine volumes and patients with elevated pH calculi were more likely to be hyperoxaluric and hypocitraturic. These findings have a number of implications. First, it is well known that adequate daily urine volume is paramount to preventing nephrolithiasis (19). Among NDMD patients who void volitionally and form primarily metabolic calculi, counseling regarding adequate hydration should be emphasized as in the non-NDMD population. A second implication regards increased risk of lithogenic factors in elevated pH stone formers; hyperoxaluria and hypocitraturia. Antibiotic exposure and hyperoxaluria due to decreased enteric oxalobacter formigenes is well documented (20, 21). Given greater risk of positive urine cultures, it is possible that these patient's microbiota are more frequently exposed to antibiotics. Hypocitraturia in this setting can be explained by degradation of citrate by uropathogens and supplementation should be considered. These data reinforce complex interactions between the host, uropathogens, and antibiotic exposure that may lead to an increase in metabolic risk factors.

Our study has some limitations. First, a small sample size has contributed to some missing data regarding stone disease and objective characterization of bladder function. Second, many patients in our database (59 / 100) lack of both a chemical analysis and 24-hour urine collection. While this can certainly contribute to selection bias, we feel that any additional published data in this complex patient population is useful. Additionally, while many patients had two 24-hour urine collections, we analyzed only the one performed closest to their first chemical stone analysis. Third, a lack of some interesting variables limit our analysis. For example, we did not collect data on history of recurrent urinary tract infection and antibiotic exposure. We did, however, report bladder management, which may be the most important risk of recurrent urinary infection. Fourth, there are too few events in this small sample to perform a reliable multivariable analysis, as at least 10 to 20 events are needed to fit one variable into a linear or logistic model. Despite this limitation, we provide important hypothesis generating results that will be a focus of future projects. We are now prospectively collecting data on this population and plan to perform more robust analyses for future studies. Finally, we did not find the expected difference in urinary pH between patients with difference stone classifications. This finding may be explained by the lack of baseline urine metabolic profile for most patients, who submitted a first 24-hour urine analysis after sterilization of their urinary tract and subsequent treatment of their stone. Future studies may clarify this trend by obtaining baseline 24-hour urine analyses in patients with NDMD.

The motivation for this study was the large number of patients with NDMD and metabolic calculi. This study supports a full metabolic evaluation and medical management in patients with neurologic deficiencies or elevated pH stones, contrary to other published literature and practices. Clearly, further study is needed to support our data and we aim to continue studying our NDMD population. Some of the most important implications of renal calculi in this population include delayed presentation and a higher complication rate during treatment. Therefore, more focus should be placed on stone prevention.

## CONCLUSIONS

Among patients with NDMD, metabolic factors may play a more significant role in renal calculus formation than previously believed. There is still a high incidence of carbonate apatite calculi, which could be attributed to bacteriuria. However, obesity, low urine volumes, hypocitraturia, and hyperoxaluria suggest an under-recognized metabolic contribution. Our results reinforce the importance of behavioral counseling and antibiotic stewardship in this population. Additionally, identifying metabolic risk factors in NDMD patients is challenging but important, as it has the potential to significantly affect recurrence and repeat urologic procedures for renal calculus disease.

## Abbreviations

NDMD: Neurologically derived musculoskeletal deficiencies
SCI: spinal cord injury
SB: spina bifida
CVA: cerebrovascular accident
CP: cerebral palsy
BMI: body mass index
